# Thermodynamic phase transitions in a frustrated magnetic metamaterial

**DOI:** 10.1038/ncomms9278

**Published:** 2015-09-21

**Authors:** L. Anghinolfi, H. Luetkens, J. Perron, M. G. Flokstra, O. Sendetskyi, A. Suter, T. Prokscha, P. M. Derlet, S. L. Lee, L. J. Heyderman

**Affiliations:** 1 Laboratory for Mesoscopic Systems, Department of Materials, ETH Zurich, 8093 Zurich, Switzerland; 2 Laboratory for Micro- and Nanotechnology, Paul Scherrer Institute, 5232 Villigen PSI, Switzerland; 3 Laboratory for Neutron Scattering, Paul Scherrer Institute, 5232 Villigen PSI, Switzerland; 4 Laboratory for Muon Spin Spectroscopy, Paul Scherrer Institute, 5232 Villigen PSI, Switzerland; 5 Sorbonne Universités, UPMC Univ Paris 06, UMR 7614, LCPMR, 75005 Paris, France; 6 CNRS, UMR 7614, LCPMR, 75005 Paris, France; 7 School of Physics and Astronomy, SUPA, University of St. Andrews, St. Andrews KY16 9SS, UK; 8 Condensed Matter Theory Group, Paul Scherrer Institute, 5232 Villigen PSI, Switzerland

## Abstract

Materials with interacting magnetic degrees of freedom display a rich variety of magnetic behaviour that can lead to novel collective equilibrium and out-of-equilibrium phenomena. In equilibrium, thermodynamic phases appear with the associated phase transitions providing a characteristic signature of the underlying collective behaviour. Here we create a thermally active artificial kagome spin ice that is made up of a large array of dipolar interacting nanomagnets and undergoes phase transitions predicted by microscopic theory. We use low energy muon spectroscopy to probe the dynamic behaviour of the interacting nanomagnets and observe peaks in the muon relaxation rate that can be identified with the critical temperatures of the predicted phase transitions. This provides experimental evidence that a frustrated magnetic metamaterial can be engineered to admit thermodynamic phases.

## Abstract (web summary)

Recently, periodic arrays of thermally active nanomagnets with bistable magnetization have been built which mimic the behaviour of frustrated magnets and model Ising systems. Here, the authors use muon spin relaxation to evidence thermodynamic phase transitions in an artificial kagome ice system.

## Graphical abstract

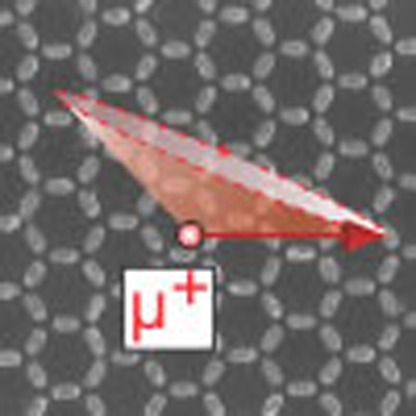

Materials with highly frustrated magnetism support multiple phases with complex magnetic structure and have provided a platform for the discovery of surprising behaviour such as the absence of long range order even at the lowest temperatures in spin ices^1^,^2^ and spin liquids^3^, and the appearance of excitations such as emergent magnetic monopoles^4^. Up to now, much of the research in this area has been driven by microscopic modelling and the behaviour of spins in bulk crystals, which often have complex microscopic interactions. Taking a different approach, one can realize speculative ideas about magnetic frustration with tailor-made materials composed of functional mesoscopic building blocks—in this case magnetic—placed in specific densely packed arrangements. These frustrated magnetic metamaterials, like their photonic counterparts^5^,^6^, offer the possibility to design and control the individual degrees of freedom in a way that can go beyond naturally occurring microscopic systems.

Here we consider a magnetic system well-known from a theoretical point of view, namely artificial kagome spin ice^7^,^8^,^9^,^10^. This two-dimensional system was originally designed to mimic the kagome phase in the pyrochlore spin ice crystals and has been predicted to support phase transitions^11^,^12^,^13^. It consists of elongated single domain nanomagnets arranged on a two-dimensional kagome lattice and coupled via their dipolar magnetic fields (see Fig. 1a). Each nanomagnet is single domain and can be represented as a single magnetic moment—or macrospin—aligned in one of two directions along the magnet long axis, effectively having Ising spin degrees of freedom. Analogous to spin ice, the specific geometry of the lattice does not allow all interactions to be simultaneously satisfied, delivering a highly frustrated magnetic system and leading to a large subset of quasi-degenerate local macrospin configurations-the spin ice manifold. On lowering the temperature, it has been predicted that the system undergoes two consecutive second-order phase transitions^12^,^13^, where the long-range dipolar interactions gradually lift the degeneracy of the spin ice manifold as visualised in Fig. 1b. At high temperature, the system remains disordered in a highly degenerate spin ice phase (Ice I) with the only constraint that, at any point in time, two macrospins point in and one points out (or *vice versa*) at each vertex. Through a first transition, the system enters into a charge-ordered phase (Ice II) and finally, through a second transition at the lowest temperature, into a long-range charge- and spin-ordered (LRO) phase. At the highest temperatures, there is a crossover to a paramagnetic regime where the system can access macrospin configurations outside the spin ice manifold. This regime should not be confused with the onset of microscopic paramagnetism in Permalloy, which is characterised by the Curie temperature that is typically >400 K and significantly larger than the temperatures considered here. We now take advantage of the distinct phase diagram of artificial kagome spin ice to demonstrate that frustrated magnetic metamaterials can support thermodynamic phase transitions equivalent to those found in a microscopic spin system.

## Results

### Artificial kagome spin ice sample design

**Figure 1:**
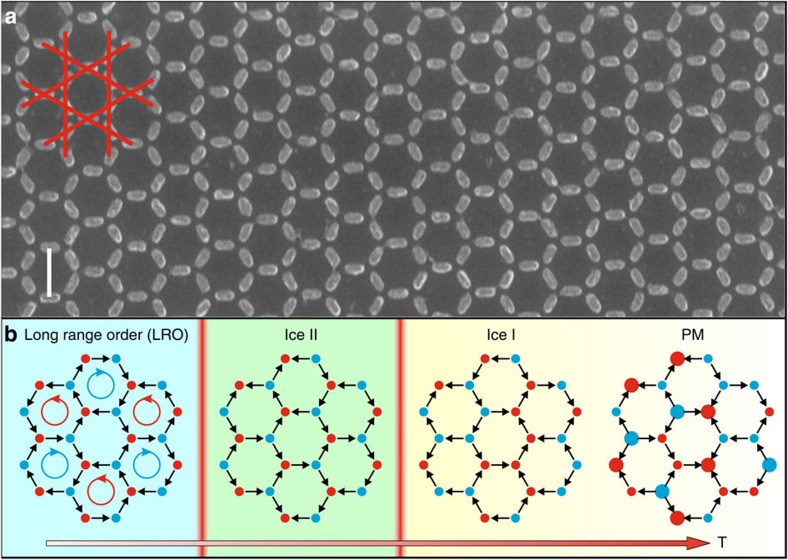
Artificial kagome spin ice and the predicted phase transitions. (**a**) Scanning electron microscope (SEM) micrograph showing a small part of an artificial kagome spin ice array with nanomagnets arranged on a kagome lattice (indicated in red). The whole array extends over an area of 25 mm^2^ and comprises 10^9^ nanomagnets. Thermally active over a wide range of temperatures, such a large system is able to emulate the thermodynamics characteristic of bulk systems. Scale bar, 170 nm. (**b**) Highlights the predicted magnetic phase transitions. Snapshots of possible spin configurations for each phase are included, with magnetic charges of opposite sign indicated in red and blue at the vertices^11^,^12^,^13^. At the highest temperatures, thermal fluctuations dominate and the system is in a paramagnetic (PM) regime. As the temperature decreases, there is a crossover to a disordered spin ice regime (Ice I) where the ice rule is strictly obeyed (2-in/1-out or 1-in/2-out moment configurations at each vertex). A first second-order phase transition (indicated with a vertical red bar) brings the system into a charge-ordered regime (Ice II). On reducing the temperature further, the system undergoes a second phase transition and enters a long range ordered (LRO) phase with both spin and charge order. The array shown in panel a corresponds to the strongly interacting sample in which the phase transitions are experimentally accessible under equilibrium conditions. The shading in panel b is a guide to the eye to identify the different phases.

We manufacture a thermally active artificial kagome spin ice system^14^,^15^,^16^, with nanomagnets having spontaneous but collective macrospin reversals, by significantly reducing the size of the magnets compared with those found in conventional artificial spin ice. Our samples are therefore constructed from nanomagnets with a length, width and thickness of 63, 26 and 6 nm, ensuring thermally activated macrospin reversals at room temperature and below. To optimize the critical temperatures of the phase transitions, we modified the inter-magnet dipolar energy scale. Specifically, we considered two samples characterized by different nanomagnet separations, *a* (see Figs 1a,3a). The crossover from the high-temperature paramagnetic phase to the lower temperature spin ice manifold was addressed with a weakly interacting sample designed with magnets far apart (*a*=400 nm), resulting in relatively low critical temperatures in the sub 10 K range. A second strongly interacting sample was designed with much more closely packed nanomagnets (*a*=170 nm, shown in Fig. 1a), resulting in higher (sub 150 K) critical temperatures, which allowed us to observe phase transitions within the spin ice manifold. The magnetic metamaterials are manufactured over unprecedented areas. They are made up of nine 5 × 5 mm^2^ arrays separated from each other by 10 μm, with each array corresponding to 10^9^ nanomagnets. This ensures the high degeneracy of states, and therefore thermodynamics, characteristic of bulk systems.

### Temperature-dependent muon spin depolarization

**Figure 2:**
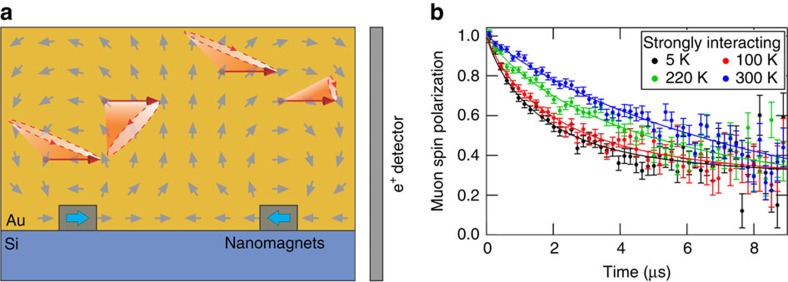
Low energy μSR experiments. A sketch of the experiments with low energy muons is shown in **a**. The 100% polarized muons (indicated by the red arrows) are implanted in the gold capping layer a few tens of nm above the nanomagnets (grey rectangles with arrows corresponding to the orientation of the macrospins) where they act as highly sensitive local magnetic probes. Here the muon spins precess (red cones) around the local magnetic field generated by the nanomagnets, resulting in a time evolution of the initial muon polarization. As the magnetic environment is inhomogeneous, different muons experience different fields and the polarization of the muon ensemble monotonically relaxes as a function of time^17^. Temporal fluctuations of the local fields due to the dynamics of the nanomagnets also lead to an experimentally observable relaxation of the muon polarization. The time-dependent polarization is determined from the muon decay by measuring the anisotropically emitted positrons (e^+^) in the surrounding e^+^ detectors. Selected muon spin relaxation (μSR) measurements of the strongly interacting spin ice sample for different temperatures are shown in **b**. The error bars represent statistical errors determined by the number of total muon decay events observed for each data point. The continuous lines are best fits to the data.

To detect effectively the occurrence of the two predicted phase transitions between Ice I and Ice II and between Ice II and LRO, we employ the well-established method of muon spin relaxation^17^ (μSR; explained in Supplementary Note 1). This technique is highly suited for measurements of weak dipolar fields generated by the nanomagnets and for the detection of the critical slowing down associated with second-order phase transitions, which is equivalent to 1/T_1_ measurements in nuclear magnetic resonance spectroscopy. In particular, μSR is a local probe of the internal magnetic fields, and is sensitive to <1 Gauss fields. In contrast to nuclear magnetic resonance, μSR measurements can be performed in true zero-applied field (ZF-μSR), so avoiding field-induced perturbations to the system, and the magnetic field dynamics can be followed in the required kHz–MHz range. The spin 1/2 low energy muons are implanted over the entire sample at normal incidence in a gold capping layer (see Supplementary Fig. 1), a few tens of nanometres above the nanomagnets, where they probe the stray fields from the nanomagnets (see Fig. 2a). Modelling each nanomagnet as a magnetic dipole, we estimate that they produce a distribution of magnetic fields, which have magnitudes of the order of 10 G, which is strong enough to affect the muon spin. Each measurement at a given temperature is performed for an ensemble of several million muons ensuring enough statistics for the measurement of the field distribution generated by the nanomagnets and a uniform examination of the entire sample.

To provide an insight into how phase transitions are detected by μSR, we begin with a qualitative description of a typical μSR experiment. The relaxation (gradual loss) of the muon spin polarization is measured as a function of time, as shown by selected ZF-μSR spectra for the strongly interacting sample in Fig. 2b. This relaxation is due to two contributions (see also Supplementary Note 2 and Supplementary Figs 2,3), the distribution (direction and magnitude) of the static local fields and their temporal fluctuations in the time window of μSR. It is the second dynamic contribution that provides the most direct and unequivocal signature of phase transitions. The two contributions result in two distinct relaxation processes visible in the μSR spectra; a fast (transverse) relaxation with the rate *λ*_T_ and a slow (longitudinal) relaxation with the rate *λ*_L_. The transverse rate is given by the distribution of static fields perpendicular to the initial muon spin direction, as well as by their temporal fluctuations, if present. The longitudinal rate is instead solely given by their temporal fluctuations. At a second-order phase transition, the relaxation time of the system diverges and crosses the time window of μSR, producing a resonance which is most directly seen as a peak of the longitudinal relaxation rate^17^,^18^,^19^,^20^. The two relaxation rates can be obtained by fitting the double-exponential model described in Equation 1 in the Methods section to the experimental data. As the temperature is increased towards high temperatures, however, the fluctuations of the local fields become increasingly faster until the muons probe a purely dynamic environment, with no static component in the μSR time window. In this fast fluctuation regime, the two relaxation rates have the same value and the relaxation of the muon spin can be approximated by a single exponential (see *T*=300 K curve in Fig. 2b). We denote the onset of the fast fluctuation regime with *T*_FF_.

**Figure 3:**
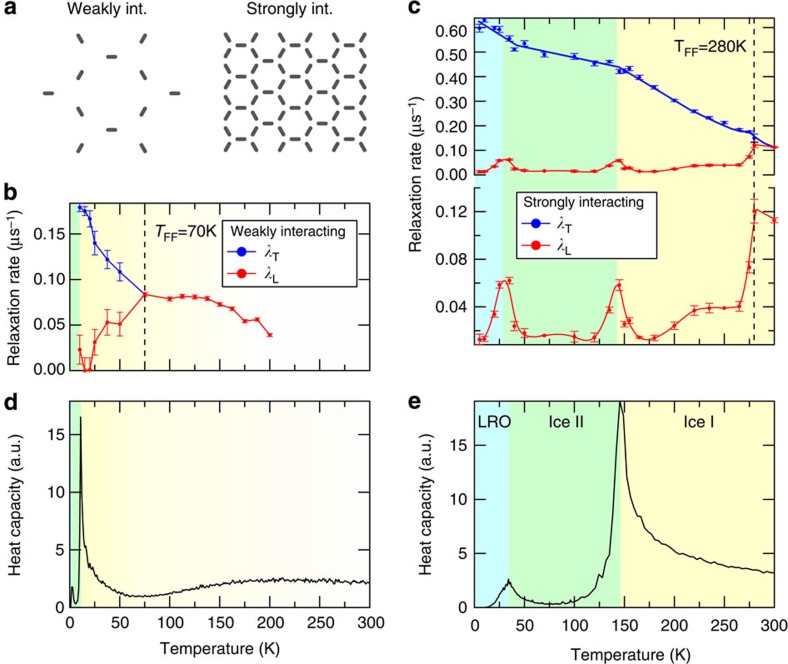
ZF-μSR relaxation rates and comparison with Monte Carlo simulations. For the weakly and strongly interacting artificial spin ice samples depicted in **a**, the relaxation rates are shown in **b** and **c**, respectively. The values of the transverse (*λ*_T_, blue) and longitudinal (*λ*_L_, red) relaxation rates were obtained by fitting a two-component relaxation function to the experimental muon spin polarization (the solid lines connecting the points in **b** and **c** are guides for the eye). The error bars represent the standard deviation of the fit parameters. For both samples, we can distinguish two main temperature regimes: at high temperatures the system is characterized by a single relaxation rate (fast fluctuation regime) and at low temperatures by two distinct rates. *T*_FF_ marks the onset of the fast fluctuation regime where *λ*_T_≈*λ*_L_. For the strongly interacting sample (**c**), *T*_FF_ is observed at a higher temperature than in the weakly interacting sample, indicating the slowing down of magnetic fluctuations due to the stronger magnetic correlations. Below *T*_FF_, additional temperature regimes, characterized by different slopes of *λ*_T_ and separated by peaks of *λ*_L_ at 145 and 35 K, can be distinguished. These peaks indicate critical fluctuations of the magnetic moments associated with the phase transitions between Ice I and Ice II at 145 K, and between Ice II and LRO at 35 K. The heat capacity computed from Monte Carlo simulations for both samples is shown in **d** and **e** (black curves). For the strongly interacting sample (**e**), the two peaks of the heat capacity occur at temperatures that match those of the phase transitions observed in the experiments. For the weakly interacting sample (**d**), the peaks in the heat capacity are shifted to lower temperatures, confirming that they are no longer experimentally accessible. The shading in **b–e** is a guide to the eye to identify the different phases. a.u., arbitrary unit; Int., interacting.

With this qualitative picture in mind, we now present μSR data from our two samples with carefully chosen nanomagnet separations that give a complete picture of the thermodynamic behaviour. We start with the temperature dependence of the two relaxation rates, *λ*_T_ and *λ*_L_, for the weakly interacting sample (Fig. 3b, Supplementary Fig. 4 and Supplementary Note 3). Since the nanomagnets are far apart, the effects of interactions only become important at very low temperatures, so that the system remains in a disordered phase down to the lowest experimentally accessible temperature. The observed behaviour is therefore similar to that of an ensemble of non-interacting superparamagnetic particles^21^, with a slowing down of the fluctuations of the macrospins with decreasing temperature. Above *T*_FF_=70 K, the system is in the fast fluctuation regime and the μSR spectra can be described by a single (dynamic) relaxation rate (red points in Fig. 3b). Below 70 K, the fluctuations become slower and the time-averaged magnetic field at the muon stopping site becomes non-zero, resulting in two distinct relaxation rates *λ*_T_ (blue points) and *λ*_L_ (red points). Therefore, the weakly interacting sample exhibits a paramagnetic behaviour down to 70 K and, as the temperature is further decreased, it develops increasingly correlated macrospin fluctuations, as reflected by the increasing separation of the transverse and longitudinal relaxation rates.

With the strongly interacting sample, which has much more closely packed nanomagnets and stronger correlations between the macrospins, we bring the phase transitions within the experimentally accessible temperature range. The relaxation rates for this sample are shown in Fig. 3c. Compared to Fig. 3b, two differences can be noted. The fast fluctuation regime now occurs at a higher temperature, above *T*_FF_=280 K, indicating that the sample is still superparamagnetic at room temperature and that magnetic correlations are indeed stronger than in the weakly interacting sample. Below *T*_FF_, the longitudinal relaxation rate *λ*_L_ has two resonance peaks at 35 and 145 K (the corresponding ZF-μSR spectra are shown in Supplementary Fig. 2) and the slope of the transverse relaxation rate *λ*_T_ is not constant but has different values for the different temperature ranges separated by the peaks. These peaks indicate dynamic processes that match the μSR timescale at these temperatures (more details about this observation can be found in the Supplementary Note 2) and provide a clear evidence for critical fluctuations that accompany magnetic phase transitions^17^,^18^,^19^,^20^.

## Discussion

We can interpret the phase transitions indicated by the peaks of the longitudinal relaxation rate for the strongly interacting sample to be the transitions between Ice I and Ice II (at 145 K) and between Ice II and LRO (at 35 K). To support this interpretation, we compared the experimental results with Monte Carlo simulations of the artificial kagome spin ice, including dipolar-like interactions up to the fifth nearest neighbours. The obtained temperature dependence of the heat capacity is shown for the weakly and strongly interacting samples in Fig. 3d,e. Consistent with previous theoretical work^11^,^12^,^13^, we find peaks in the heat capacity associated with the phase transitions between the Ice I, Ice II and the LRO phases. For the weakly interacting sample, the two peaks in the heat capacity occur at lower temperatures, which is compatible with the relaxation data of Fig. 3b.

Our observation of thermodynamic phase transitions in macroscopically large artificial kagome spin ice sets the stage for the discovery of further thermodynamic phenomena in frustrated magnetic metamaterials, in particular exotic magnetic phases in systems that need not have an equivalent in nature. While theory strongly suggests the nature of the phase transitions in artificial spin ice materials, recent developments in synchrotron X-ray scattering methods^22^ may make it feasible to obtain complementary information on the spatial correlations that correspond to the phase transitions experimentally revealed using muon spin spectroscopy.

## Methods

### Sample fabrication

The samples were fabricated by electron beam lithography. A polymethyl methacrylate polymer layer was spin coated on a silicon substrate and then exposed with a Vistec EBPG 5000Plus electron beam writer. A 6 nm-thick Permalloy (Ni_80_Fe_20_) film was subsequently deposited by thermal evaporation and capped with 2 nm of gold to prevent oxidation. As discussed in Supplementary Note 4,5 and Supplementary Fig. 5, the volume of the nanomagnets is small enough to ensure thermally activated macrospin reversals within the timescale of μSR at all investigated temperatures. Following lift-off, the patterned nanomagnet array was coated with a continuous film of 80 nm of gold to stop the muons ∼30 nm above the nanomagnets. We verified the absence of muon diffusion in this layer up to 300 K (see Supplementary Note 6 and Supplementary Fig. 6).

### Experiments

The μSR experiments have been performed at the low-energy muon beamline^23^,^24^,^25^ of the Swiss Muon Source as described in the Supplementary Note 1. The experiments were carried out in zero applied field in the temperature range between 300 and 5 K. The sample was oriented so that the initial spin of the muons was parallel to one set of nanomagnets. At each temperature, 7.5 million muon decay events were counted over a time of ∼50 min.

### μSR model

As the distribution of local fields generated by the nanomagnets in the Au capping layer is not trivial, we considered an empirical model based on exponentials, which includes both static and dynamic effects. The muon polarization is fitted using the MUSRFIT^26^ software package and described by the following equation


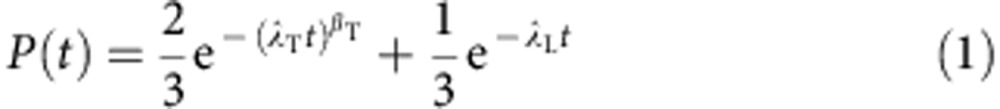


The first term accounts for the transverse spin relaxation and the second term accounts for the longitudinal relaxation. The stretching exponent *β*_T_ describes the shape of the field distribution, and we obtain a value of 0.83 below the fast fluctuation regime and 1.0 in the fast fluctuation regime, respectively. The validity of our model has been confirmed with a model-independent inverse Laplace analysis^27^,^28^,^29^ of the experimental data (see the Supplementary Note 2).

### Simulations

We used Monte Carlo simulations to compute the equilibrium distribution of the macrospin configurations. Details of the kagome ice model are discussed in the Supplementary Note 7. Peaks in the temperature dependence of the heat capacity reflect the critical behaviour associated with the phase transitions predicted to occur in this system^12^,^13^.

## Additional information

**How to cite this article:** Anghinolfi, L. *et al*. Thermodynamic phase transitions in a frustrated magnetic metamaterial. *Nat. Commun.* 6:8278 doi: 10.1038/ncomms9278 (2015).

## Supplementary Material

Supplementary Information Supplementary Figs 1-6, Supplementary Notes 1-7 and Supplementary References.
