# Supplementary material for: Thermodynamic phase transitions in a frustrated magnetic metamaterial
Source: Nat Commun. 2015 Sep 21;6:8278. doi: 10.1038/ncomms9278 (PMC4595626; doi:10.1038/ncomms9278)
Supplement: Supplementary Information — Supplementary Figs 1-6, Supplementary Notes 1-7 and Supplementary References. [file ncomms9278-s1.pdf]

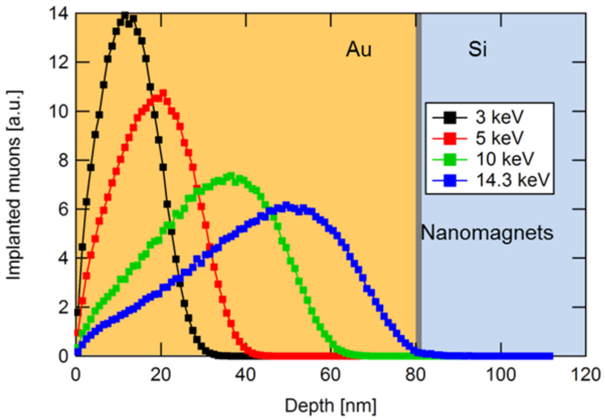

Supplementary Figure 1: Muon stopping profile in the Au capping layer of the artificial spin ice samples. By setting the kinetic energy of the incoming muons, it is possible to tune their average stopping depth and therefore their average distance from the nanomagnets (indicated with a grey line) on the Si substrate. For our experiments we used a muon energy of 14.3 keV, corresponding to a majority of muons stopping between 20 nm and 40 nm above the nanomagnets. This depth was chosen to stop the muons in a region where the magnetic stray fields originating from the nanomagnets are strong enough to influence the muon precession.

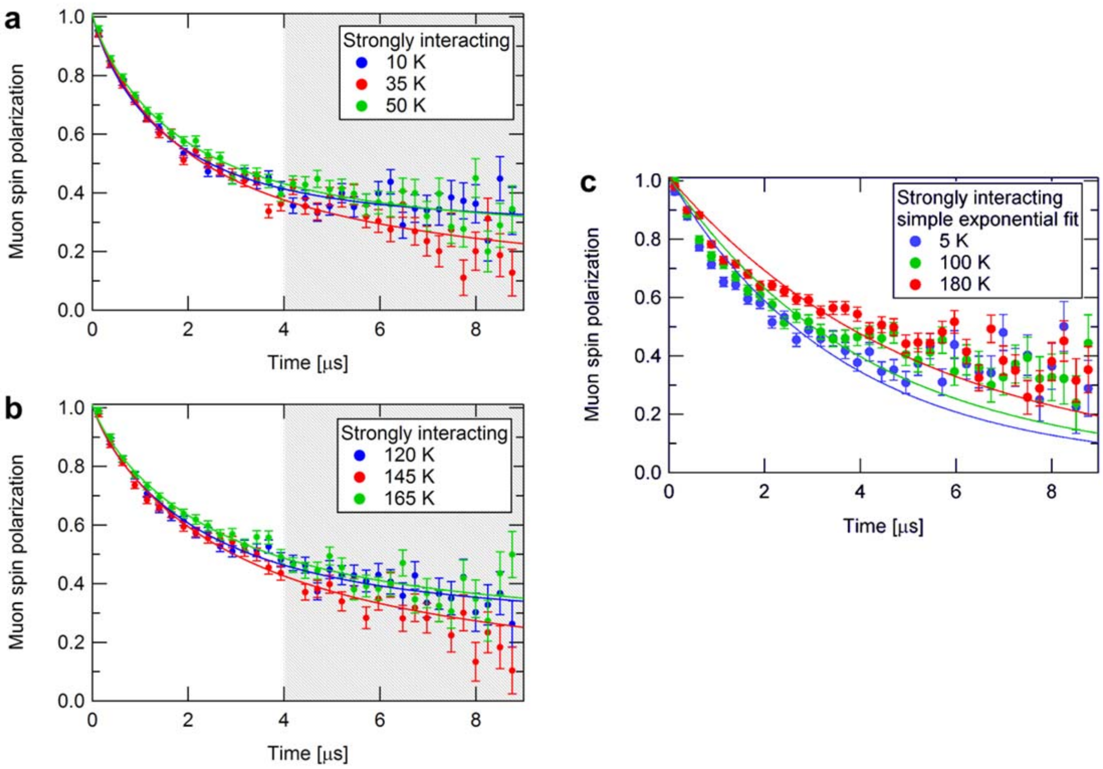

Supplementary Figure 2: Selected ZF-µSR spectra for the strongly interacting sample. Panel a, b: ZF-µSR spectra at temperatures close to the peaks of the longitudinal relaxation rate at 35 K and 145 K, respectively. As explained in the main text, the muon spectra are characterized by a fast relaxation (related to the distribution of both static and dynamic fields) followed by a slower relaxation in the tail at long times (related to dynamics only, shaded regions). Away from the peaks (blue and green points), the tail of the spectra is almost constant indicating that the characteristic time scale of the fluctuations of the local magnetic fields does not match the µSR time scale. However, close to the critical temperatures (red points) the long-time spectrum more strongly relaxes. This is an unequivocal signature of dynamic local fields in the time window of µSR. It should be noted that this effect, which is already clearly visible in the ZF-µSR spectra, is then reflected in the peaks of the longitudinal rate λ_L at the magnetic phase transitions. In panel c, we show simple exponential functions fitted to the experimental data. The fits fail to reproduce the ZF-µSR spectra both at short times and at long times, further confirming that a two-component relaxation function is required to analyse the data. In all panels, the error bars represent statistical errors determined by the number of total muon decay events observed for each data point.

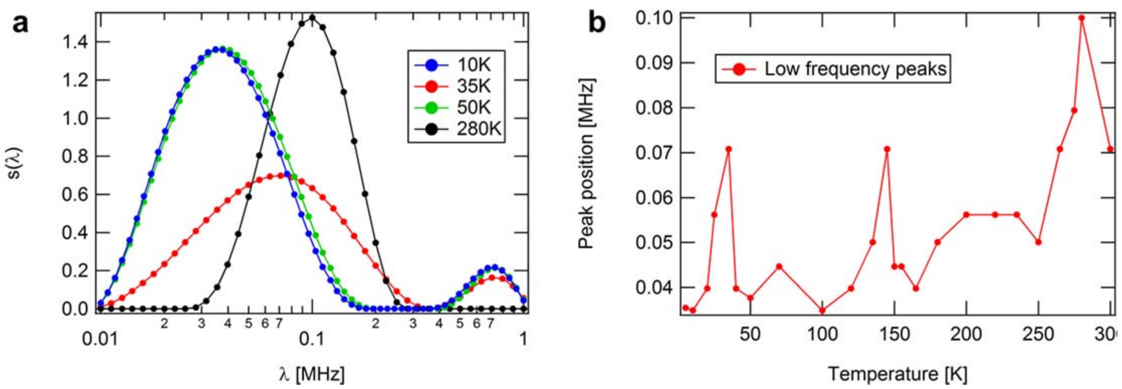

Supplementary Figure 3: Inverse Laplace transformation of the ZF-μSR data of the strongly interacting sample. Panel a: Below 280 K the spectrum has a bimodal distribution of relaxation rates independently confirming, both qualitatively and quantitatively, the analysis with the two-component relaxation function. The distribution centred around the lower relaxation rates corresponds to the longitudinal rate λ_L from the time-domain fit, while the distribution centred around the higher relaxation rates is associated with the transverse relaxation rate λ_T. At the magnetic phase transition temperature of 35 K, λ_L increases due to critical fluctuations in the time window of μSR. In the fast fluctuation regime above 280 K, the bimodal distribution $s(\lambda)$ collapses to a unimodal distribution in which λ_T and λ_L are no longer distinguishable. Panel b: The position of the maximum of the lower distribution of relaxation rates as a function of temperature reproduces the same behaviour as the longitudinal relaxation rate, with two peaks at 35 K and 145 K.

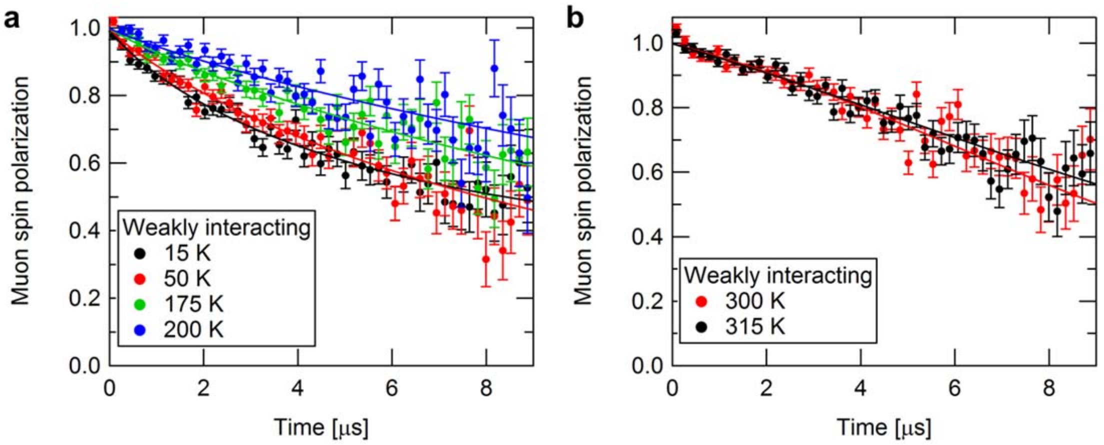

Supplementary Figure 4: Selected μSR spectra measured on the weakly interacting sample. Panel a shows spectra below 200 K which were analysed with a two-component relaxation function. The spectra shown in panel b were measured above 200 K and can be described by a Gaussian Kubo-Toyabe relaxation function[1]. In both panels, the error bars represent statistical errors determined by the number of total muon decay events observed for each data point and the continuous lines are best fits to the data.

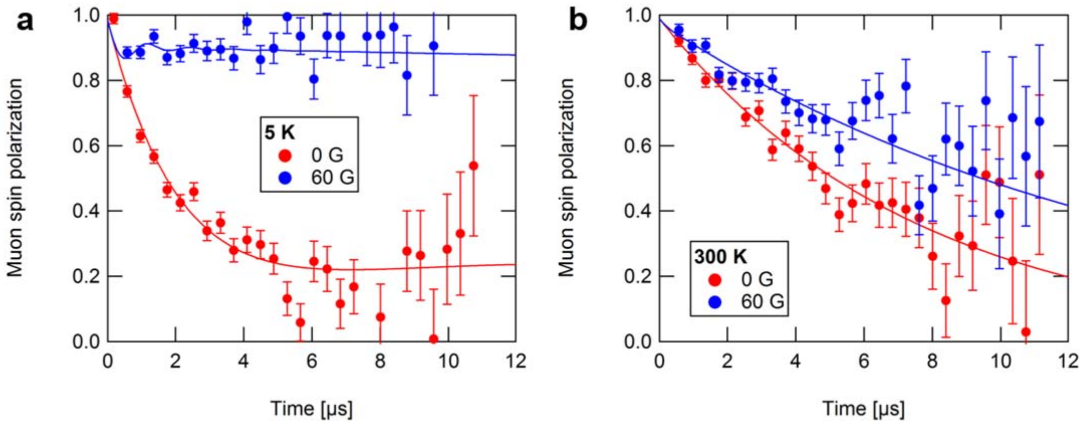

Supplementary Figure 5: Longitudinal field μSR measurements for the strongly interacting sample. Muon spin relaxation spectra measured (a) at 5 K and (b) 300 K in zero field (red points) and on application of a 60 G longitudinal field normal to the sample plane and parallel to the initial muon spin direction (blue points). The continuous lines are the corresponding best fit curves using a dynamic Lorentzian Kubo-Toyabe function. These data confirm that the fields at the muon sites are static at 5 K while they are fluctuating with a rate of approximately 3 MHz at 300 K. In both panels, the error bars represent statistical errors determined by the number of total muon decay events observed for each data point.

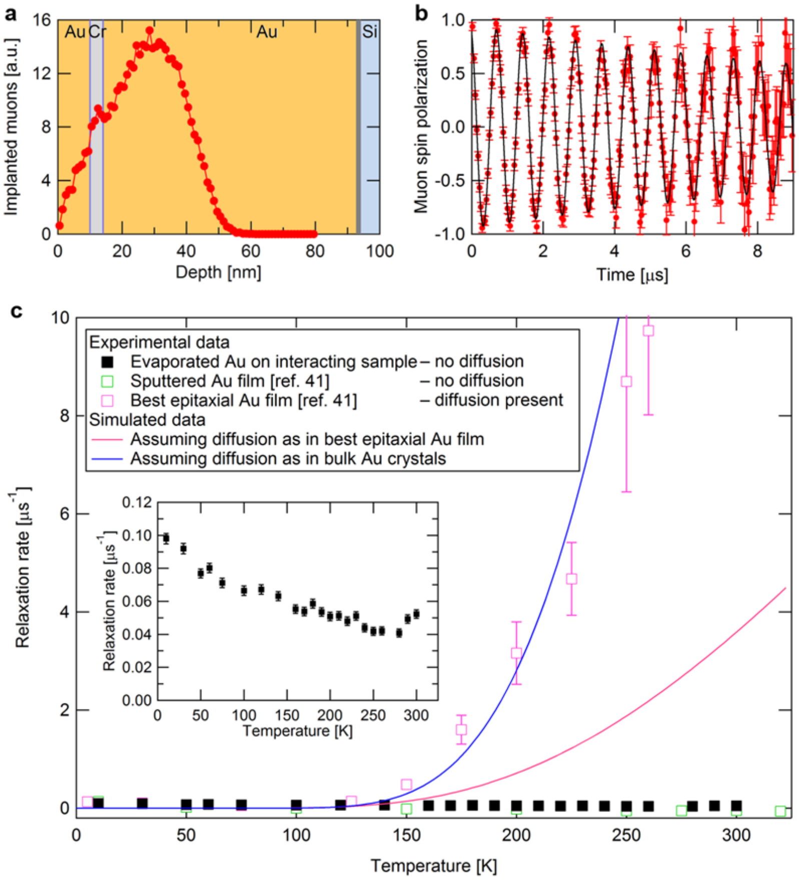

Supplementary Figure 6: Validation of absence of muon diffusion in the gold capping layer. Panel a: muon stopping profile computed with the Monte Carlo code TRIM.SP[2] for the strongly interacting sample after evaporation of 4 nm of Cr and 10 nm of Au. Panel b: muon spin rotation in a 100 G transverse field at 300 K. The error bars represent statistical errors determined by the number of total muon decay events observed for each data point. The continuous line is the best fit to the data (see text for details). Panel c: relaxation rate λ measured in the evaporated Au capping layer of the strongly interacting sample (black points), in a sputtered Au film (without muon diffusion)[3] and in an epitaxial Au film (with muon diffusion)[3]. The continuous lines are the relaxation rates expected if the muons would diffuse in the Au layer on top of the nanomagnets, using the parameters for Au films estimated in ref. 3. The relaxation rate in our sample (highlighted in the inset) is in comparison small at all temperatures and does not increase with temperature, verifying that the muons do not diffuse in our sample at all investigated temperatures. The error bars represent the fit errors.

Supplementary Note 1. Low energy μSR instrumentation and muon stopping ranges

Muon spin relaxation (μSR) employs spin-polarized positive muons as local magnetic probes to study the magnetic properties of solid state matter. Conventional μSR uses a beam of high energy (> 4 MeV) muons, with a stopping range of a fraction of a millimetre, and is suited for the investigation of bulk materials. In low energy μSR, the muon energy is reduced to keV's, allowing the study of thin films or heterostructures as a function of the muon implantation depth on the nm scale[4]. A detailed description of the low energy μSR setup can be found on the website of the LEM group at Paul Scherrer Institute (http://www.psi.ch/low-energy-muons/lem-low-energy-muons-group).

The key component of the Low Energy Muons (LEM) beamline at the Paul Scherrer Institute is a condensed layer of solid N_2 or Ar,[5] which moderates muons with 4 MeV energy to a kinetic energy of about 15 eV without affecting the initial spin polarization[6]. Re-accelerating these low energy muons by means of an electrostatic transport system results in a beam of ≈100% spin polarized muons with a tuneable energy between 0.5 and 30 keV, and with a characteristic implantation depth between a fraction of nm and several hundred nm. The actual stopping range profile can be calculated by the Monte Carlo program TRIM.SP[2]. For example, the stopping profiles for our artificial spin ice samples capped with an 80 nm layer of Au are shown in Supplementary Figure 1 for different muon energies.

The implanted muon thermalizes at an interstitial lattice site within a few picoseconds. Here the muon spin interacts with its magnetic environment and the spin polarization of the muon ensemble $P(t)$ relaxes as a function of time. In a radioactive beta decay, the muon decays via the process $\mu^+ \rightarrow e^+ + \bar{\nu}_\mu + \nu_e$. The positron is finally detected by a pair of plastic scintillators located to the left and right side of the sample (see Figure 2a). Due to parity violation in the μ^+ decay process, the positron is emitted preferentially along the direction of the muon spin at the moment of the decay. By collecting several millions of positron counts in the detector pair, it is therefore possible to reconstruct the time evolution of the muon spin polarization, which can be analysed to obtain information about the static and dynamic magnetic properties of the sample.

Supplementary Note 2. μSR data analysis

For a detailed introduction to the analysis of μSR data, the interested reader is referred to the recent textbook of Yaouanc and Dalmas de Réotier[7]. The analysis of the time-dependent μSR data has been performed using the free software package MUSRFIT[8]. In a magnetic powder or in situations where the internal fields at the muon sites are randomly oriented for the muon ensemble, the muon polarization function $P(t)$ is given by a sum of two contributions. The so-called transverse relaxation function comprises 2/3 of the spectrum originating from local magnetic fields perpendicular to the initial muon spin and the longitudinal fraction accounts for the remaining 1/3 of the spectrum with the internal field parallel to the initial muon spin. We have therefore fitted the following polarization function to the data:

$$P(t) = \frac{A}{A_0} \cdot \left(\frac{2}{3} \mathrm{e}^{-(\lambda_\mathrm{T} t)^{\beta_\mathrm{T}}} + \frac{1}{3} \mathrm{e}^{-\lambda_\mathrm{L} t} \right), \quad (1)$$

where A is the measured decay asymmetry and A_0 is the total asymmetry. In addition, a non-relaxing background contribution due to muons stopping in the Ag sample holder has been subtracted from the data. The relaxation rate of the transverse component λ_T is governed by the distribution of static fields sensed by the muon ensemble as well as the relaxation due to dynamic fluctuations of the magnetic fields[7], if present. The longitudinal relaxation rate λ_L is solely given by magnetic dynamics in the time window of μSR. λ_L is therefore always smaller (or equal) to λ_T. In the fast fluctuation regime both rates become equal and are therefore no longer distinguishable. As the distribution of local fields generated by the nanomagnets in the Au capping layer is not trivial, we considered an empirical model based on a stretched exponential function with the exponent β_T. Stretched exponentials are commonly used to describe the muon depolarization in the presence of a distribution of relaxation rates. They are also quite flexible functions, which can fit a wide range of line shapes. Firstly the β_T parameter has been kept as a free fit parameter and found to be nearly temperature independent for temperatures below 280 K. In the final analysis, β_T has been fitted to one common parameter for all datasets below 280 K and found to be $\beta_\mathrm{T} = 0.83$. In the fast fluctuation regime, β_T was found to approach a value close to 1.0 as expected. The validity of our fitting model (Supplementary Equation 1) has been confirmed with a model-independent inverse Laplace analysis of the experimental data (see below). In Supplementary Figure 2, selected μSR spectra are shown, which are either below (blue), at (red) or above (green) the two observed magnetic phase transitions. It is already clearly visible from this raw

data that, at the transitions, the long-time tail of the spectrum (highlighted by grey-shading in Supplementary Figure 2) more strongly relaxes (red curves) than for the data taken at temperatures away from the transitions, where the long-time tail is essentially flat (green and blue curves). The relaxation in the long-time tail gives peaks in the fit parameter λ_L at the magnetic phase transitions, see Figure 3c. As mentioned above, the non-zero longitudinal relaxation rate of the peaks λ_L indicates that the timescale of the fastest magnetic fluctuations crosses the time window of μSR, which naturally happens at second order phase transitions[7, 9].

In order to validate that the model fits the μSR spectra (see Supplementary Equation 1), we used CONTIN[10, 11, 12] to compute the inverse Laplace transform of the experimental data. The algorithm decomposes the raw μSR data into a series of exponentials $s(\lambda) \cdot e^{-\lambda t}$ (where t is time and λ an inverse of time and, in our specific case, a relaxation rate) and computes the distribution $s(\lambda)$ of their amplitudes, which satisfies Supplementary Equation 2:

$$P(t) = \int_{\lambda_{\min}}^{\lambda_{\max}} s(\lambda) \mathrm{e}^{-\lambda t} \mathrm{d}\lambda, \qquad (2)$$

In contrast to the Fourier transform, the Laplace inversion does not have a unique solution. Our aim here is therefore to check that Supplementary Equation 1 is compatible with the family of solutions $s(\lambda)$ generated by the algorithm.

In Supplementary Figure 3, we show representative solutions computed by CONTIN at key temperatures. The solutions for the spectra measured at 10 K and 50 K (blue and green points, i.e. below and above the transition at 35 K) indicate a bimodal distribution of relaxation rates, a narrow one centred at 0.03 μs^{-1} and a broader one centred at 0.7 μs^{-1} (please note the logarithmic scale). This bi-modal distribution established using a model-independent approach confirms that the two-component relaxation function in Supplementary Equation 1 is appropriate for the analysis of the μSR data in the time domain, where the narrow low frequency distribution corresponds to the simple exponential for the longitudinal relaxation rate and the second broader distribution corresponds to the stretched exponential for the transverse relaxation rate. The relaxation rates obtained from the inverse Laplace analysis also compare quantitatively very well with the values of $\lambda_L = 0.01$ μs^{-1} and $\lambda_T = 0.6$ μs^{-1} obtained at 10 K and 50 K with the model in Supplementary Equation 1.

Looking at the solution for the data at the magnetic phase transition at 35 K (red points in Supplementary Figure 3), we see that the narrow distribution at small λ is now at 0.08 μs^{-1}, corresponding to the relaxation rate of 0.06 μs^{-1} at the top of the first peak in Figure 3c, and

providing a model-independent confirmation of the enhancement of magnetic fluctuations in the time window of μSR at the phase transition. Finally, in the fast fluctuation regime at 280 K (black points in Supplementary Figure 3), the solution becomes unimodal centred around 0.1 μs^{-1} in agreement with a single relaxation rate of 0.12 μs^{-1} obtained from the time-domain fit (Figure 3c). The match between the two independent analysis methods, on in the time domain and one in the inverse-time (frequency) domain, is therefore excellent and fully supports the original choice of the two-component relaxation function to describe the data.

Supplementary Note 3. Selected μSR spectra measured for the weakly interacting sample

In Supplementary Figure 4a, selected ZF-μSR spectra of the weakly interacting sample that were also analysed with Supplementary Equation 1 are shown (solid lines). Above 200 K (see curves in Supplementary Figure 4b), magnetic fluctuations in the nanomagnets are too fast to be seen in the time-window of the technique. Instead, the spectrum can be described by a Gaussian Kubo-Toyabe function[1], which is typical for a random field distribution generated by nuclear magnetic moments in the vicinity of the muon. The Gaussian shape of the spectrum indicates that the magnetic fields at the muon sites are static on the timescale of the measurement. By fitting the spectra with the Gaussian Kubo-Toyabe function[1], we obtain a Gaussian relaxation rate of $\sigma = (0.045 \pm 0.004)$ MHz, in agreement with the small nuclear magnetic moment of Au. In addition, these measurements also indicate that, even up to highest measured temperatures, the muons do not diffuse through the gold layer. In order to be sure that muon diffusion does not contribute to the high temperature data, dedicated measurements were performed on the gold layer as described in Section 1.4.

Supplementary Note 4. Longitudinal field μSR measurements

The zero field μSR measurements presented in the main article indicate that there is mainly static magnetic behaviour at low temperatures with the observation of clearly distinct transverse and longitudinal relaxation rates λ_T and λ_L. With increasing temperature, λ_T decreases while λ_L increases until both relaxation rates converge to the same single rate in the fast fluctuation regime. In the absence of muon diffusion, as verified in Section 1.4, a non-zero longitudinal relaxation rate allows for no other interpretation than dynamic

magnetic fields at the muon sites indicating fluctuations of the nanomagnet magnetization in the time window of μSR[7].

To further verify that the magnetic fields seen by the muons are dynamic at high temperatures and static at low temperatures, we performed longitudinal field (LF) μSR measurements on the strongly interacting sample, which allows us to discriminate between static and dynamic fields at the muon site[7]. Using the recently installed spin rotator of the LEM beamline[13], we rotated the polarization of the incoming muons compared to the zero field measurements, orienting it perpendicular (instead of parallel) to the sample surface. Then we measured the muon spin relaxation in zero field and in a longitudinal field (parallel to the muon spin) of 60 G. The obtained spectra for 5K and 300 K are shown in Supplementary Figure 5. The zero field spectra are consistent with the data presented in the main article (Figure 2b), where the initial muon spin direction was oriented in the film plane. Specifically, at 5 K (red curve in Supplementary Figure 5a), the polarization exhibits an initially fast relaxation characterized by λ_T, which is followed by an essentially constant tail ($\lambda_L \approx 0$). At 300 K (red curve in Supplementary Figure 5b), the ZF spectrum can be described by a single exponential relaxation function as appropriate for the fast fluctuation regime.

On applying a longitudinal field at 5K (blue curve in Supplementary Figure 5a), we find that the initial muon spin polarization is now essentially conserved over time. This behaviour is a result of the fact that the applied field is much larger than the dipolar fields from the nanomagnets and the dipolar fields are static on the muon timescale[7]. In contrast, at 300 K the applied field has very little influence on the time dependence of the muon spin polarization (compare the red curve with the blue curve in Supplementary Figure 5b). In particular, the fact that the spectrum still relaxes provides unequivocal evidence for magnetic fields which are dynamic in the time window of μSR[7].

To estimate the fluctuation rate of the dipolar fields from the nanomagnets, we fitted the muon spin polarization data with a dynamic Lorentzian Kubo-Toyabe function[14, 15], which accounts for the longitudinal applied field and local field dynamics in the strong collision approximation. The best-fit curves are plotted in Supplementary Figure 5 as continuous lines. At 300 K, the fluctuation rate is determined to be 2.9 MHz, which is comparable with the μSR timescale and therefore consistent with the onset of the fast fluctuation regime at 280 K. In contrast, at 5 K the fluctuation rate is found to be zero within the fit error. This confirms the presence of dynamic local fields at high temperatures in the fast fluctuation regime and

the presence of static local fields at low temperatures deduced from the ZF measurements presented in the main article.

Supplementary Note 5. Estimation for nanomagnet moment reorientation timescale

In ref. 16, a barrier energy of 0.925 eV and a prefactor of 10^{12} sec^{-1} were found to describe the dynamics of moment reorientation of an individual nanomagnet reasonably well. Such a barrier energy was also comparable to that resulting from the shape anisotropy. In ref. 16 the volume of a nanomagnet was $470\times170\times3$ nm^3, which is much larger than that used presently ($63\times26\times6$ nm^3). Assuming the barrier energy is due to shape anisotropy (which is proportional to magnet volume), this gives a barrier energy estimate for our current system of approximately 40 meV. Using the same prefactor of 10^{12} sec^{-1}, and assuming a simple Arrhenius form, at 40 K (the temperature regime of the lowest critical temperature) the timescale between nanomagnet reorientations is of the order of 0.1 nanoseconds, which is well within the timescale of the muon precession and quite compatible with the temperature scale of the assumed blocking temperature of an isolated nanomagnet. It is emphasized that all of these numbers are order of magnitude estimates.

Supplementary Note 6. Absence of muon diffusion in the evaporated Au overlayer

In μSR, a finite longitudinal relaxation rate λ_L is due to the temporal fluctuation of the magnetic field experienced by the individual implanted muons. In principle, this can have two different origins. Either the local magnetic field at the muon changes its direction as a function of time (dynamic magnetism) or the muon moves due to thermally activated diffusion between interstitial lattice sites in the background of an otherwise static magnetic environment (muon diffusion). These two effects are difficult to disentangle without *a priori* knowledge of the material under investigation.

In the present study, we investigate the magnetic properties of the artificial kagome spin ice system by implanting the muons in an Au capping layer and measuring static and/or dynamic magnetic fields generated by the stray fields of the nanomagnets. For interpreting

the high temperature data correctly, it is important to check whether or not the muon diffuses through the gold layer in the investigated temperature range.

Previous measurements on a high quality bulk Au crystal revealed long range (nm) thermally activated muon diffusion above approximately 100 K[17]. Performing a systematic study on thin metallic films[3] with the same low energy muon apparatus at the Paul Scherrer Institute used in the present work, it was also found that in the best quality epitaxial Au films muon diffusion sets in at approximately 100 K. Nevertheless, it was demonstrated that sputtered films showed no muon diffusion up to 300 K, indicating that the diffusion appears to only occur in high quality crystalline materials.

In the present study, the Au capping layers were prepared by thermal evaporation, which results in polycrystalline films. Therefore we can expect that muon diffusion is also suppressed in our samples. Nevertheless, we verified this assumption by actually measuring the diffusion in the same strongly interacting sample used for the investigation presented in the main article. Here we used a similar method as shortly described in ref. 3 and explained in detail in ref. 18.

We applied the following procedure to measure the muon diffusion in our sample. After carrying out the main experiments, we deposited a thin film (4 nm) of antiferromagnetic Cr on top of the thick (80 nm) Au capping layer and capped it with a second thin film of Au (10 nm). Then we measured the time evolution of the muon spin polarization of muons initially implanted close to the Au/Cr interface, mainly in the thick Au layer, using 8 keV muons (the stopping profile is shown in Supplementary Figure 6a). If the muons were to diffuse, they would reach the Cr film where they would quickly depolarize. The time-dependent reduction of the muon polarization therefore reflects the quantity of muons reaching the interface to the Cr layer. The faster the muons diffuse, the faster is the observed relaxation of the μSR signal. If the muons do not diffuse, only a very slow depolarization will be observed. The measured relaxation rate λ can be related to the microscopic diffusion parameters[18]:

$$\lambda = \frac{\pi^2}{(2a)^2}\frac{d^2}{6}\,\Gamma_0 \exp\left(-\frac{E_\mathrm{a}}{k_\mathrm{B}T}\right), \quad (3)$$

with a being the distance between the maximum of the muon stopping profile and the Cr layer, $d = 0.288$ nm the jump distance between two octahedral interstitial lattice sites in Au where the muon is hopping, Γ_0 the attempt frequency, and E_a the activation energy. Supplementary Equation 3 has been derived for Cr layers on both sides of the Au film. For

our geometry with a single Cr layer, we can expect the relaxation rate to be approximately a factor of two smaller.

We measured the relaxation rate by μSR in a weak transverse field of 100 G, which was applied perpendicular to the film and the initial muon spin direction. A representative μSR spectrum is shown in Supplementary Figure 6b. The oscillatory component is due to the precession of the muon spin around the applied field and the muon spin polarization function $P(t)$ can be fitted with the following function:

$$P(t) = A/A_0 \cos(\omega_\mu t + \phi) \mathrm{e}^{-\lambda t}, \quad (4)$$

where A_0 is the maximally observable asymmetry of the muon decay, ω_μ is the Larmor precession frequency ($\omega_\mu = 2\pi \cdot 13.55\ kHz\ G^{-1} \cdot 100\ G$), ϕ the initial phase and λ the relaxation rate. An additional term for a non-relaxing background contribution due to the Ag sample plate has also been taken into account. The resulting values of λ as a function of temperature are shown in Supplementary Figure 6c as solid black data points. The relaxation rate is found to be small at all temperatures and does not increase with temperature, verifying that the muons do not diffuse in our sample at all investigated temperatures. The slight decrease of the relaxation rate as a function of temperature stems from the weak stray fields of the nanomagnets, which are probed by the muons at a much larger distance compared to the measurements presented in the main article. For comparison, the diffusion measurement data[3] for a sputtered Au film (green points), in which the muons also do not diffuse, and one epitaxial Au film (pink points), in which the strongest diffusion has been observed, are shown in the same figure. In addition the dashed lines represent expected relaxation rates for our geometry if the muon were to diffuse as in bulk Au samples[17] ($\Gamma_0 = 10^{13.5}$Hz, $E_a/\ k_\mathrm{B} =\ 1350$ K) or as in the best epitaxial sample[3] ($\Gamma_0 = 10^{12.1}$Hz, $E_a/k_\mathrm{B} = 970$ K). Clearly, the simulated curves for muon diffusion are far from the measured data.

In conclusion, we have confirmed that there is no long range (nm) muon diffusion in the Au capping layer of our samples in the measured temperature range between 5 K and 300 K. This absence of diffusion is also in accordance with the observation of a Gaussian-like nuclear relaxation at high temperatures in the weakly interacting sample (Supplementary Figure 4b), which at the same time also excludes other types of diffusion, such as grain boundary diffusion at high temperatures.

Supplementary Note 7. Monte Carlo Simulations

We used Monte Carlo simulations to compute the equilibrium configurations of the nanomagnet moments. The conventional way to model the individual nanomagnets in artificial spin ice is to approximate them as single Ising macrospins arranged on a finite kagome lattice[16, 19]. The approximation is justified because the nanomagnets are small enough to be single-domain and the thickness of few nm's ensures that the exchange interactions between the microscopic spins dominate, keeping them aligned parallel to each other. Nevertheless, it has recently been shown using micromagnetic simulations[20] that, when the nanomagnets are brought close together, the magnetization at the tips of the nanomagnets slightly curls to minimize the dipolar interactions. As a consequence, and also due to the spatial extent of the nanomagnets, the short range interactions between nanomagnets are enhanced. In our simulations, we have effectively accounted for these effects by following an approach similar to Budrikis and coworkers[19]. We employed the macrospin model on a kagome lattice and considered modified interactions, J_n, between n-th nearest-neighbours $<i,j>_n$. The Hamiltonian is the following:

$$H = \sum_{n=1}^{5} J_n \sum_{<i,j>n} \frac{1}{r_n^3} \left[\mathbf{m}_i \cdot \mathbf{m}_j - 3(\mathbf{m}_i \cdot \mathbf{r}_{ij})(\mathbf{m}_j \cdot \mathbf{r}_{ij})\right], \quad (5)$$

where $\mathbf{m}_i$ is the macrospin unit vector for the magnet i , $\mathbf{r}_{ij}$ is the unit vector connecting the magnets i and j, and r_n is the distance between the n-th nearest-neighbours. Interactions up to fifth nearest-neighbours were required to obtain the low temperature transition to the LRO state. Fitting the numerical values of the interaction parameters to the phase transition temperatures of the experimental data gives J_1 = 5.5, J_2 = 4.8, J_3 = 4.8, J_4 = 4.8, J_5 = 4.8, in units of $J_0 = \mu_0 m_0^2/4\pi$, the value expected for unmodified point dipole interactions with $m_0 = 2.7 \times 10^{-18}$ A·m^2 measured with a SQUID magnetometer. As expected, for the fitted values of J, the balance between short and long range interactions is modified in favour of the short range interactions ($J_1 > J_2, J_3, J_4, J_5$) and the values of J indicate that in the simulation, the magnitude of the magnetic moment ($=\sqrt{4.8}\, m_0$) is comparable to the experimental value. The simulations were carried out on an array containing N=2646 nanomagnets and by varying the temperature from 300 K down to 5 K in steps of 1 K. The same set of parameters was used for both samples, the only difference being the lattice constant. For each temperature, we performed 1000 Monte Carlo sweeps to obtain a well-converged heat capacity, which gives direct information on the fluctuations in the system internal energy at a particular temperature. Here a Monte Carlo sweep is defined as N single site Monte Carlo steps

followed by $N/2$ hexagonal loop Monte Carlo steps, where the latter loop algorithm was the same as that used in ref. 21.

References

1. Kubo R, Toyabe T. Magnetic resonance and relaxation; proceedings of the XIVth Colloque Ampere Ljubljana, 6-11 September 1966. (North-Holland Pub. Co., 1967).

2. Eckstein W. *Computer Simulation of Ion-Solid Interactions*. (Springer Berlin Heidelberg, 1991).

3. Luetkens H*, et al.* Diffusion of muons in metallic multilayers. *Physica B* **326**, 545-549 (2003).

4. Morenzoni E, Prokscha T, Suter A, Luetkens H, Khasanov R. Nano-scale thin film investigations with slow polarized muons. *J Phys-Condens Mat* **16**, S4583-S4601 (2004).

5. Prokscha T*, et al.* The new mu E4 beam at PSI: A hybrid-type large acceptance channel for the generation of a high intensity surface-muon beam. *Nucl Instrum Meth A* **595**, 317-331 (2008).

6. Morenzoni E*, et al.* Generation of Very Slow Polarized Positive Muons. *Phys Rev Lett* **72**, 2793-2796 (1994).

7. Yaouanc A, Dalmas de Réotier P. *Muon spin rotation, relaxation, and resonance applications to condensed matter*. (Oxford University Press, 2011).

8. Suter A, Wojek BM. musrfit: a free platform-independent framework for muSR data analysis. *Physcs Proc* **30**, 69-73 (2012).

9. Slichter CP. *Principles of magnetic resonance*, 3rd edn. (Springer, 1990).

10. Provencher SW. Inverse Problems in Polymer Characterization - Direct Analysis of Polydispersity with Photon Correlation Spectroscopy. *Makromol Chem* **180**, 201-209 (1979).

11. Provencher SW. A Constrained Regularization Method for Inverting Data Represented by Linear Algebraic or Integral-Equations. *Comput Phys Commun* **27**, 213-227 (1982).

12. Provencher SW. Contin - a General-Purpose Constrained Regularization Program for Inverting Noisy Linear Algebraic and Integral-Equations. *Comput Phys Commun* **27**, 229-242 (1982).

13. Salman Z*, et al.* Design and Simulation of a Spin Rotator for Longitudinal Field Measurements in the Low Energy Muons Spectrometer. *Physcs Proc* **30**, 55-60 (2012).

14. Hayano RS, Uemura YJ, Imazato J, Nishida N, Yamazaki T, Kubo R. Zero-Field and Low-Field Spin Relaxation Studied by Positive Muons. *Phys Rev B* **20**, 850-859 (1979).

15. Dalmas de Réotier P, Yaouanc A. Quantum Calculation of the Muon Depolarization Function - Effect of Spin Dynamics in Nuclear Dipole Systems. *J Phys-Condens Mat* **4**, 4533-4556 (1992).

16. Farhan A*, et al.* Exploring hyper-cubic energy landscapes in thermally active finite artificial spin-ice systems. *Nat Phys* **9**, 375-382 (2013).

17. Brown JA*, et al.* Muon Depolarization by Paramagnetic Impurities in Nonmagnetic Metals. *Phys Rev Lett* **47**, 261-264 (1981).

18. Luetkens H. Magnetism of thin films and multilayers investigated by low energy muon spin rotation. (Technical University of Braunschweig, Germany, 2004).

19. Budrikis Z*, et al.* Domain dynamics and fluctuations in artificial square ice at finite temperatures. *New J Phys* **14**, 035014 (2012).

20. Rougemaille N*, et al.* Chiral nature of magnetic monopoles in artificial spin ice. *New J Phys* **15**, 035026 (2013).

21. Moller G, Moessner R. Magnetic multipole analysis of kagome and artificial spin-ice dipolar arrays. *Phys Rev B* **80**, 140409(R) (2009).
